# Assessing the Therapeutic Efficacy of Proton Transport Inhibitors in a Triple-Negative Breast Cancer Murine Model with Magnetic Resonance Imaging—Chemical Exchange Saturation Transfer Tumor pH Imaging

**DOI:** 10.3390/metabo13111161

**Published:** 2023-11-18

**Authors:** Chetan Dhakan, Annasofia Anemone, Vittoria Ventura, Antonella Carella, Alessia Corrado, Elisa Pirotta, Daisy Villano, Feriel Romdhane, Francesco Gammaraccio, Silvio Aime, Dario Livio Longo

**Affiliations:** 1 Institute of Biostructures and Bioimaging (IBB), National Research Council of Italy (CNR), Via Nizza 52, 10126 Turin, Italy; 2 Department of Molecular Biotechnology and Health Sciences, University of Turin, Via Nizza 52, 10126 Turin, Italy; 3 IRCCS SynLAB SDN, Via Gianturco 113, 80143 Naples, Italy

**Keywords:** breast cancer, proton pump inhibitor, 4T1, triple-negative breast cancer, pH imaging, chemical exchange saturation transfer (CEST), magnetic resonance imaging (MRI), acidosis, tumor

## Abstract

Proton transporters play a key role in maintaining the acidic tumor microenvironment; hence, their inhibition has been proposed as a new therapeutic treatment, although few methods can accurately assess their effect in vivo. In this study, we investigated whether MRI-CEST (Magnetic Resonance Imaging—Chemical Exchange Saturation Transfer) tumor pH imaging can be a useful tool to evaluate in vivo the therapeutic efficacy of several Proton Pump Inhibitors (PPIs) in breast cancer. Cell viability and extracellular pH assays were carried out in breast cancer cells cultured at physiological pH (7.4) or acid-adapted (pH of 6.5 and 6.8) following the exposure to inhibitors of V-ATPase (Lansoprazole, Esomeprazole) or NHE1 (Amiloride, Cariporide) at several concentrations. Next, triple-negative breast cancer 4T1 tumor-bearing mice were treated with Lansoprazole or Amiloride and MRI-CEST tumor pH imaging was utilized to assess the in vivo efficacy. Only Lansoprazole induced, in addition to breast cancer cell toxicity, a significant inhibition of proton extrusion. A significant reduction in tumor volume, prolonged survival, and increase in extracellular tumor pH after 1 and 2 weeks were observed after Lansoprazole treatment, whereas no significant changes were detected upon Amiloride treatment. Our results suggested that MRI-CEST tumor pH imaging can monitor the therapeutic efficacy of PPIs in breast cancer murine models.

## 1. Introduction

Breast cancer is one of the most commonly diagnosed and leading cause of cancer death in women. Despite improvements in the early detection, advanced breast cancer with distant organ metastases is still considered incurable and remains a great health threat to women worldwide [1,2]. Therefore, it is essential to develop new therapeutic agents to non-invasively detect and treat metastatic breast cancer. As compared to other forms of breast cancer, triple-negative breast cancer (TNBC) has a more aggressive clinical course, increased tendency to yield visceral metastases, and significantly lower survival rate with limited treatment options [3,4]. Solid tumors often show an acidic tumor microenvironment (TME) that reflects dysregulated physiological and metabolic conditions with reduced oxygen (hypoxia) and glucose concentrations and correspondingly increased H^+^ and lactate levels [5,6,7,8,9]. This leads to enhanced acidification of the extracellular TME with pH in the range of 6.5–6.8, which in turn leads to higher invasiveness and metastasis [10,11,12,13,14,15]. In order to maintain a slightly alkaline intracellular pH, tumor cells must expel the excess of protons throughout trans-membrane proton transporters. Actually, a large array of transporting proteins such as H^+^/K^+^-ATPase [16], Na^+^/H^+^ exchanger (NHE1) [17,18,19], vacuolar ATPase (V-ATPase) [20,21], monocarboxylate transporters (MCTs) [22,23], carbonic anhydrase (CAs) [24,25], or bicarbonate transporters are overexpressed or up-regulated [26]. The occurrence of acidic pH in the TME has suggested therapeutic procedures based on the inhibition of proton transporters to suppress cancer cell growth and metastases formation [27,28,29,30,31,32,33]. Two of the most studied proton transporters in breast cancer are V-ATPases and NHE1, whose aberrant activity contributes to create a reversed pH gradient that promotes mammary tumor growth and invasiveness [34,35,36]. Recent evidence has demonstrated that the inhibition of V-ATPase activity by Lansoprazole leads to cytosolic acidification and intracellular reactive oxygen species accumulation, increasing the therapeutic efficacy of doxorubicin in breast cancer cells both in vitro and in vivo [37,38]. Furthermore, Amiloride, an NHE1 inhibitor, demonstrated a marked antitumor activity when combined with cisplatin in 4T1 triple-negative breast tumors, whereas, when used in combination with celecoxib, it significantly reduced the incidence rates of pulmonary metastases from a primary rat mammary carcinoma [39].

The need of in vivo assessment of tumor pH has prompted the development of several imaging methods and pH-responsive probes for measuring the tumor pH [40]. Among the different imaging modalities, Magnetic Resonance Imaging (MRI) can provide accurate assessment of the anatomy and of other microenvironmental properties of the mammary glands [41,42,43,44,45]. More recently, the MRI—Chemical Exchange Saturation Transfer (MRI-CEST) technique has been demonstrated as a robust approach for an accurate determination of tumor pH by exploiting the exchange of saturated mobile protons of pH-sensitive agents with the bulk water pool for generating an MRI-visible pH-dependent contrast [46,47,48,49,50,51,52]. In particular, iopamidol-based tumor pH imaging can deliver an accurate and excellent spatial resolution of in vivo tumor acidosis in several cancers [53,54,55,56,57,58]. Notably, this approach first provided in vivo evidence of the relationship between dysregulated metabolism and tumor acidosis [55] and of the association of tumor acidity with increased invasiveness [12]. Moreover, MRI-CEST tumor pH imaging is emerging as an interesting biomarker for assessing the in vivo response to several anticancer therapies, including drugs targeting proton transporters in brain tumors [59,60] and in prostate cancers [61,62]; monocarboxylate transporters in breast cancer, pharyngeal squamous cell carcinoma [63], and brain tumors [64]; pyruvate dehydrogenase kinase in breast cancer [65]; mammalian target of rapamycin complex 1 (mTORC1) in pancreatic cancer and mantle cell lymphoma cancer [66]; or mitochondrial pyruvate carrier in breast cancer [67].

In the present study, we investigated the antitumor properties of V-ATPase and NHE1 inhibitors in two aggressive breast cancer cell lines and their effect on the extracellular tumor pH. According to our knowledge, this is the first study where MRI-CEST tumor pH imaging has been exploited to assess the in vivo efficacy of PPI in the TNBC 4T1 murine model.

## 2. Materials and Methods

### 2.1. Chemicals and Reagents

Lansoprazole, Esomeprazole, Amiloride, and Cariporide (Sigma-Aldrich, St. Louis, MO, USA) were dissolved in DMSO and then re-suspended at 75 mM, 70 mM, 400 mM, and 250 mM, respectively, in RPMI 1640 medium with FBS, glutamine, streptomycin, and penicillin (Lonza, Basel, Switzerland). Iopamidol was kindly provided by Bracco Imaging (Milan, Italy).

### 2.2. Cell Culture

4T1 cells, a BALB/c-derived mouse mammary carcinoma corresponding to stage IV of human breast cancer, were obtained from American Type Culture Collection (ATCC LGS standards, Sesto San Giovanni, Italy). TS/A breast cancer cells, derived from a spontaneous BALB/c mammary tumor, were kindly provided by Prof. Federica Cavallo (University of Turin) [68]. Both 4T1 and TS/A cells were grown in RPMI-1640 medium supplemented with 10% fetal bovine serum (FBS), 100 U/mL penicillin, 100 μg/mL streptomycin (Pen/Strep), and 2 mM L-glutamine. The cells were grown at 37 °C in a humidified atmosphere containing 5% CO_2_. Additionally, 4T1 cells were also grown in RPMI-1640 modified medium (Sigma-Aldrich, St. Louis, MO, USA) supplemented with 25 mM HEPES, 25 mM PIPES, 10%FBS, 100 U/mL (Pen/Strep), and 2 mM L-Glutamine and with sodium bicarbonate to adjust pH to the values of 7.4, 6.8, and 6.5. The cells were progressively adapted to grow in the acidic culture medium; the adopted procedure took about four to five weeks.

### 2.3. Cell Proliferation/Cell Viability Assay

4T1 cells were seeded at 1.0 × 10^4^ cells, whereas TS/A cells were seeded at 5.0 × 10^3^ cells per well in 96-well Nunc-Immuno Microwell plate in buffered RPMI-1640 and unbuffered RPMI-1640 (*w*/*o* sodium bicarbonate) adjusted at pH 7.4, and at pH of 6.8 and 6.5 for 4T1 cells, and after an overnight incubation, they were treated with Lansoprazole (20, 50, 150 µM), Esomeprazole (35, 70, 140 µM), Amiloride (100, 400, 800 µM), and Cariporide (100, 250, and 500 µM), respectively, for an additional 24 h. Next, cell-containing media were added with 0.5 mg/mL 3-(4,5-Dimethyl-thiazol-2-yl)-2,5-diphenyltetrazolium bromide (MTT) and, after 4 h of incubation at 37 °C, formazan crystals were formed, which were dissolved in 100 µL of DMSO solution. Absorbance was measured at 570 nm using a spectrometer (BioTek Instruments Inc., Winooski, VT, USA) with a 96-well plate reader. All experiments were run in triplicate wells and repeated at least twice.

### 2.4. Expression of V-ATPase Western Blot

Protein extraction was performed using RIPA lysis buffer (Merk Millipore #20-188, Merck Life Sciences S.r.l., Milano, Italy), supplemented with protease inhibitor cocktail (Sigma #P2714, St. Louis, MO, USA). Lysates were centrifuged for 20 min at 13,200 RPM at 4 °C, and protein concentrations were determined using Thermo Fisher Pierce BCA protein assay kit (Thermo-Fisher, #23225, Waltham, MA, USA). An amount of 30 μg total protein was separated using Bio-Rad Mini-PROTEAN^®^ TGX TM Gel (Bio-rad #456-9034, Segrate, Italy) using Tris/glycine/SDS running buffer). Proteins were transferred to a 45 μm pore polyvinylidene difluoride (PVDF) membrane (Immobilon PSQ, Millipore, Merck-Millipore, Milano, Italy) and the membrane was blocked in TBS-T (Tris-buffered saline with 0.1% Tween-20) with 5% milk. Primary antibodies for ATP6V1A (1:1000; Abcam #137574, Cambridge, UK) and β-actin (1:3000; Sigma-Aldrich #A1978, St. Louis, MO, USA) were detected using anti-rabbit IgG (1:5000; Sigma-Aldrich #A6154, St. Louis, MO, USA) and anti-mouse IgG (1:5000; Sigma #A4416, St. Louis, MO, USA). Signals were developed using Pierce TM ECL Western blotting substrate kit (Thermo-Fisher #32106, Waltham, MA, USA). Densitometric analyses were carried out using the ImageJ software (version 1.53c).

### 2.5. In Vitro Extracellular pH Measurements

4T1 cells were seeded at 8.0 × 10^4^ cells per well in a 96-well Nunc-Immuno Microwell plate reader for 24 h. After 24 h of incubation, the plate was then treated with different concentrations of Lansoprazole (20, 50, 150 µM), Esomeprazole (35, 70, 140 µM), Amiloride (100, 400, 800 µM), and Cariporide (100, 250, and 500 µM), for an additional 24 h. For the pH-Xtra Glycolysis Assay (Agilent, Santa Clara, CA, USA), 4T1 cells were first incubated under CO_2_-free conditions and 95% humidity at 37 °C, for ~2.5 h prior to measurement. Meanwhile, pH-Xtra reagent was reconstituted in 1 mL of deionized water, and then warmed to 37 °C. Respiration buffer was made by dissolving the respiration buffer tablet in 50 mL of deionized water, the pH was adjusted to 7.4, and then the solution was filter-sterilized. Cells in the microplate were carefully washed with respiration buffer. An amount of 90 μL of pre-warmed respiration buffer was added to each well, and the plate was placed on the plate heater to equilibrate it to 37 °C. The plate was then read in a pre-heated Gen5 plate reader (BioTek Instruments Inc., Winooski, VT, USA) using dual read TR-F (Time Resolved-Fluorescence measurement) at 360 ± 40 nm (excitation) and at 620 ± 10 nm (emission) wavelengths.

### 2.6. Tumor Xenograft Animal Model

**Figure 1:**
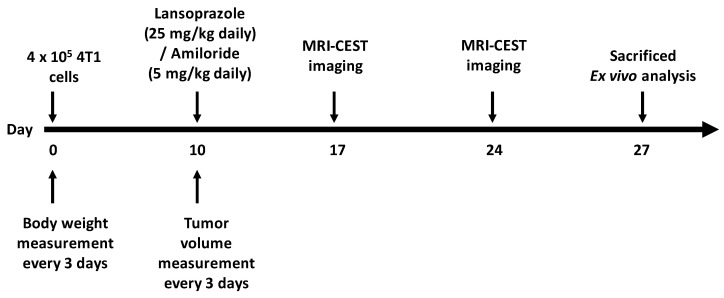
Schematic representation of the timeline of the in vivo study.

Female BALB/c mice, 6 to 8 weeks old, were purchased from Charles River Laboratories (Calco, Italy). Mice were subcutaneously implanted with 4.0 × 10^5^ 4T1 cells in both flanks with a 27-gauge insulin syringe. When mice showed signs of palpable tumor (~5 mm^3^), mice were divided into two cohorts. First cohort was divided into two groups, consisting of the control (n = 6) and Lansoprazole-treated groups (n = 6) receiving Lansoprazole administered i.p. 25 mg/kg four times a week. The second cohort consisted of the control (n = 6) and Amiloride-treated groups (n = 6) receiving Amiloride i.p. 5 mg/kg/day. All mice were randomized at the time when treatments started to avoid bias in measurements. Every 2–3 days, two perpendicular diameters of tumors were measured using Vernier caliper and body weight was also measured. Tumor volume (V) was estimated using the following formula, V = A × B^2^⁄2, where A and B are the longest and the shortest diameters, respectively. Measurements were carried out until tumors reached a maximum diameter of 800 mm^3^, when mice were killed humanely through cervical dislocation and survival curves were recorded. Schematic representation of the in vivo study is shown in Figure 1.

### 2.7. MRI-CEST Tumor pH Imaging and Analysis

MR images were acquired on a 7T Avance Neo Micro-imaging Bruker scanner (Bruker Biospin, Ettlingen, Germany) equipped with a 30 mm 1H quadrature coil. Before imaging, mice were anesthetized by injecting a mixture of xylazine 5 mg/kg (Rompun, Bayer, Milan, Italy) and tiletamine/zolazepam 20 mg/kg (Zoletil 100, Virbac, Milan, Italy) and respiratory rate was continuously monitored using an air pillow (SA Instruments, Stony Brook, NY, USA). The tail vein was cannulated with a catheter to administer iopamidol through a 27-gauge needle. After the scout image acquisition, T_2w_ anatomical images were acquired using a RARE sequence (TR = 4 s, TE = 3.7 milliseconds, NA =1, slice thickness = 1.5 mm, field of view = 30 × 30 mm^2^, matrix size = 256 × 256). Tumor pH was quantified using MRI-CEST pH imaging acquisitions [69]. CEST images were acquired before and after iopamidol i.v. injection (dose of 4 g I/kg b.w., kindly provided by Bracco Imaging SpA, Milano, Italy) using a fast multi-slice single-shot sequence preceded by a continuous-wave saturation pulse (power: 3 µT; duration: 5 s) covering the whole tumor (TE = 3.77 ms, TR = 12 s, slice thickness = 1.5 mm, number of slices = 8, Field of View = 30 × 30 mm^2^, matrix size = 128 × 128) [70]. The total acquisition time for each CEST volume was, ca., 9 min.

CEST images were analyzed using homemade MATLAB scripts (Mathworks Inc., Natick, MA, USA). Briefly, Z-spectra were interpolated using cubic smoothing splines and saturation transfer effects were calculated at 4.2 and at 5.5 ppm, respectively, and post-contrast ST maps were subtracted to pre-contrast ST ones, to obtain the corresponding ST contrast difference (ST) maps for removing the endogenous contribution. The pixel-by-pixel extracellular tumor pH (pHe) maps were calculated upon using the ratio between the two contrast difference maps at 4.2 and at 5.5 ppm, as previously described [69]. In addition, the acidity score was calculated for taking into account the heterogeneity of pHe distribution values within the tumor region. The acidity score ranged from 1 (less acidic) to 3 (more acidic), describing tumor regions with different acidosis levels.

### 2.8. Histological Analysis

After the end of this study, mice were anaesthetized and sacrificed through cervical dislocation and mammary tumors were harvested and fixed overnight in 4% formaldehyde, following embedding in paraffin, and were longitudinally sectioned at 5 µm thickness. Images were taken using an Olympus BX53F microscope (Leica Microsystems, Tokyo, Japan) and were analyzed using ImageJ software (https://imagej.nih.gov/ij/index.html, version 1.53, accessed on 15 March 2021).

### 2.9. Statistical Analysis 

Data are expressed as mean ± SD. The significance of results was determined using unpaired Student’s *t*-test, and 1-way and 2-way ANOVA with Bonferroni multiple comparisons test. The Shapiro–Wilk test was performed and revealed that the data showed a normal distribution. All statistical evaluations were performed using Graphpad Prism version 7.0 (GraphPad Inc., San Diego, CA, USA).

## 3. Results

### 3.1. Lansoprazole and Amiloride Halt Cell Proliferation in 4T1 Metastatic Breast Cancer

**Figure 2:**
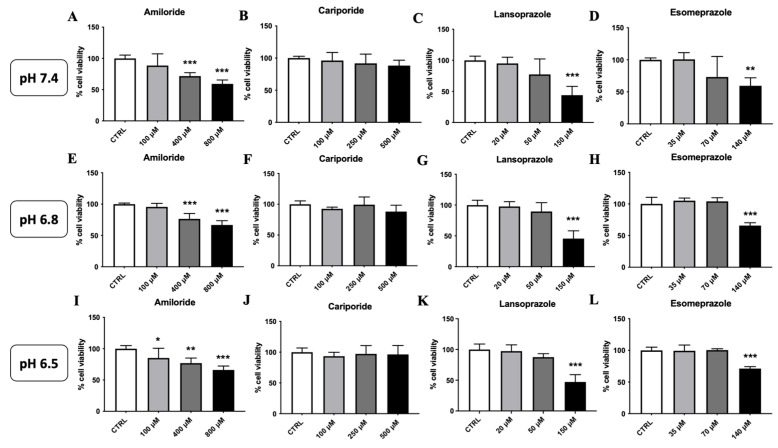
In cellulo toxicity of V-ATPase and NHE1 inhibitors on 4T1 breast cancer cell lines. Cell viability of 4T1 breast cancer cells on cells at pH 7.4 (**top**) and acid-adapted media at pH 6.8 (**middle**) and pH 6.5 (**bottom**) after 24 h of treatment with NHE1 inhibitors Amiloride (**A**,**E**,**I**) and Cariporide (**B**,**F**,**J**) or with V-ATPase inhibitors Lansoprazole (**C**,**G**,**K**) and Esomeprazole (**D**,**H**,**L**) (* *p* < 0.05; ** *p* < 0.01; *** *p* < 0.001, one-way ANOVA with post hoc Bonferroni multiple comparison test).

We first examined the anti-proliferative effect of various PPIs on two aggressive and highly metastatic breast cancer cells, 4T1 and TS/A, namely, Amiloride and Cariporide as NHE1 inhibitors and Lansoprazole and Esomeprazole as V-ATPase inhibitors. 4T1 mouse mammary carcinoma cells were treated for 24 h with increasing concentrations of PPIs in buffered media at pH 7.4 as well as in 4T1 cells adapted to grow at the acidic pH of 6.8 and of 6.5. The latter experiments aim at assessing possible effects on PPIs ascribable to the acidic culture medium, i.e., at conditions that mimic those occurring in the extracellular tumor microenvironment, on PPIs. Within the NHE1 inhibitors, Amiloride exhibited a marked dose-dependent cell death in 4T1 breast cancer cells in all media (Figure 2A,E,I). On the other hand, Cariporide did not show any anti-proliferative effects (Figure 2B,F,J). Within the class of V-ATPase inhibitors, Lansoprazole showed cell toxicity but only at the highest concentration with comparable toxicity effects in the acid-adapted cells at pHs of 6.8 and 6.5 (Figure 2C,G,K). Esomeprazole showed a comparable potency to the one found for Lansoprazole, with anti-proliferative effects observed only at the highest concentration in all the three media (Figure 2D,H,L).

The TS/A breast cancer cells showed comparable results upon treatment with PPIs, with Amiloride showing a concentration-dependent cell toxicity, whereas Cariporide was not effective as for the 4T1 TNBC cells (Supplementary Figure S1A,B). Both Lansoprazole and Esomeprazole showed a similar cell toxicity but only at the highest concentration (Supplementary Figure S1C,D).

### 3.2. Lansoprazole Induces a pH Increase in the Extracellular Medium of 4T1 Metastatic Breast Cancer Cells

In order to evaluate whether the inhibitory effects on the transport of the protons can modify the acidification of the extracellular space, we measured the extracellular medium pH upon PPI exposure. 

**Figure 3:**
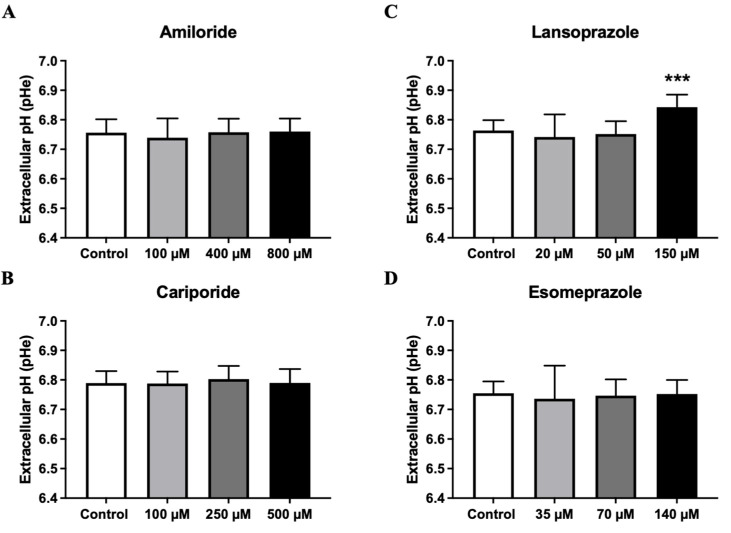
Extracellular medium pH measurements 24 h after exposure of 4T1 breast cancer cells at increasing concentrations of Amiloride (**A**), Cariporide (**B**), Lansoprazole (**C**), and Esomeprazole (**D**) in normoxic condition (*** *p* < 0.001, one-way ANOVA with Bonferroni post hoc multiple comparison test).

Amiloride, Cariporide, and Esomeprazole (Figure 3A–C, respectively) did not show any changes in the extracellular medium pH at any concentration. On the contrary, Lansoprazole induced a marked and statistically significant pH increase of the extracellular medium but only at the highest concentration (Figure 3D). Therefore, only Lansoprazole was effective in both inducing 4T1 cancer cell toxicity and reducing extracellular medium acidification. 

### 3.3. Effect of Lansoprazole on the Expression of V-ATPase

**Figure 4:**
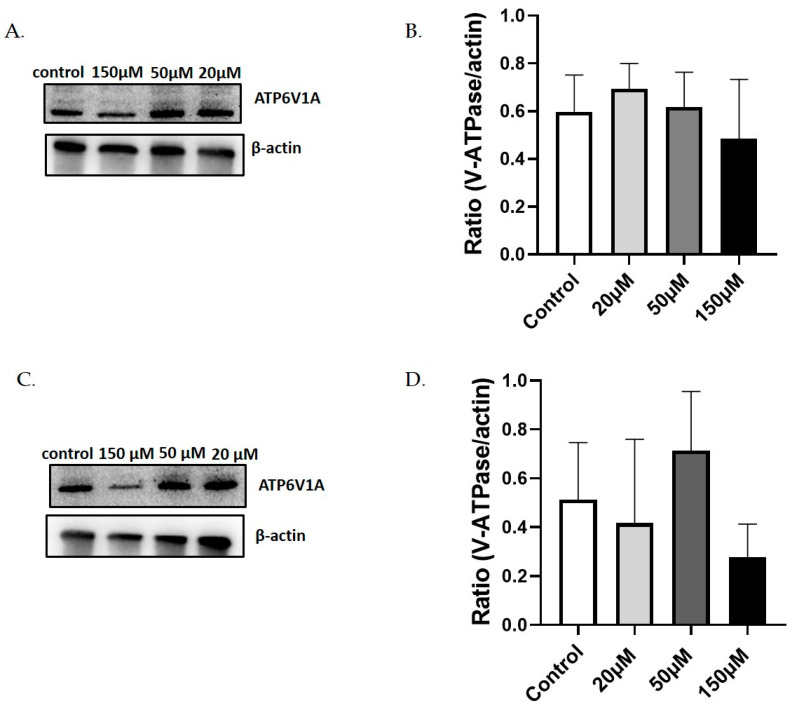
Western blot analysis and quantification of V-ATPase protein in 4T1 cells treated with different concentrations of Lansoprazole (20–50–150 µM) at pH 7.4 (**A**,**C**) or acid-adapted at pH 6.8 (**B**,**D**).

We also evaluated the effect of Lansoprazole treatment in the V-ATPase expression in the 4T1 TNBC cell line at pH 7.4 and in the corresponding acid-adapted ones at pH 6.8 through Western blot analysis (Supplementary Figure S2). The obtained results showed a modest decrease in the expression of the V-ATPase protein in 4T1 cells at pH 7.4 (Figure 4A,C) and a moderate decrease in expression, although not statistically significant, in 4T1 cells adapted at acidic pH of 6.8 (Figure 4B,D) when treated with the highest Lansoprazole concentration.

### 3.4. Lansoprazole Suppresses the Growth of Breast Carcinoma in Mice

**Figure 5:**
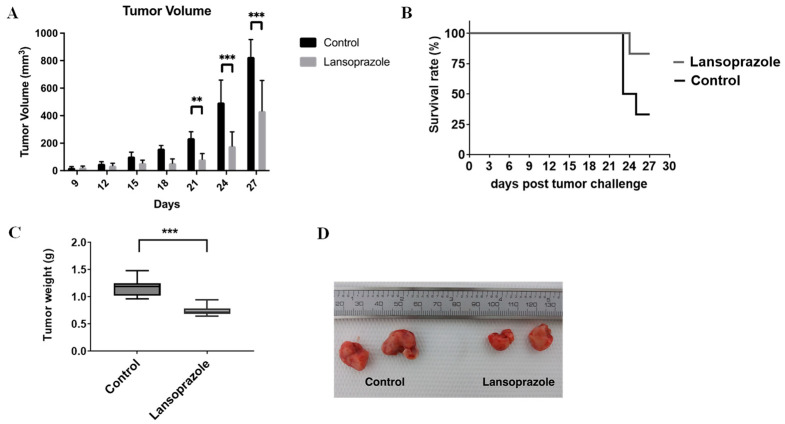
Lansoprazole suppresses tumor growth in vivo. 4T1 cells were injected into the mammary fat pads of Balb/c mice and tumors (6 for each group) were allowed to develop for 8 days. Subsequently, Lansoprazole (25 mg/kg) or vehicle was administered i.p. four days per week for 27 days. Tumor volume (**A**) was measured every three days (** *p* < 0.01; *** *p* < 0.001). Survival curves upon Lansoprazole treatments up to 27 days post-4T1 inoculation (**B**). Tumors were excised and weighed (**C**). Representative images of tumors for control and treated groups (**D**).

To assess the anti-neoplastic role of Lansoprazole in vivo, 6–8-week-old female BALB/c mice were subcutaneously administered with 4T1 breast cancer cells and, when mice showed signs of palpable tumors, they were treated with Lansoprazole (25 mg/kg/day i.p.) or with saline (control group). A significantly marked reduction in tumor volume following treatment with Lansoprazole was observed after 1 and 2 weeks when compared to untreated mice (Figure 5A). The survival curve revealed that Lansoprazole-treated mice survived longer than the control mice (statistically significant with *p* = 0.02, Figure 5B). Tumors were excised and a significant reduction in the tumor weight was observed in Lansoprazole-treated mice in comparison to control mice (1.16 g ± 0.15 vs. 0.74 g ± 0.09, *p* < 0.001, Figure 5C,D). The hematoxylin and eosin stain of tumor sections revealed a similar necrosis score and area of necrosis independent of the applied treatment (Supplementary Figure S3).

### 3.5. Lansoprazole Exhibits Increased Extracellular pH In Vivo Assessed through MRI-CEST pH Imaging

**Figure 6:**
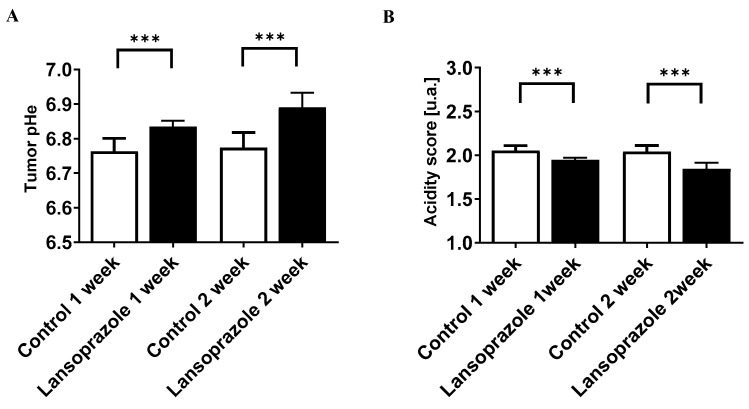
Effects of Lansoprazole (V-ATPase inhibitor) on tumor extracellular pH (pHe) and acidity score assessed using MRI-CEST. (**A**) Tumor extracellular pH (pHe) changes measured after 1 and 2 weeks of treatment. (**B**) Tumor acidity score changes calculated after 1 and 2 weeks of treatment. *** *p* < 0.001, unpaired t test.

When extracellular tumor pH was evaluated through MRI-CEST tumor pH imaging, a significant change in the extracellular pH (pHe) was observed after 1 week of Lansoprazole treatment in the TNBC 4T1 murine model in comparison to the control group (pHe = 6.83 ± 0.02 and 6.76 ± 0.03, for control and Lansoprazole-treated groups after 1 week, respectively, *p* < 0.001, Figure 6A). 

**Figure 7:**
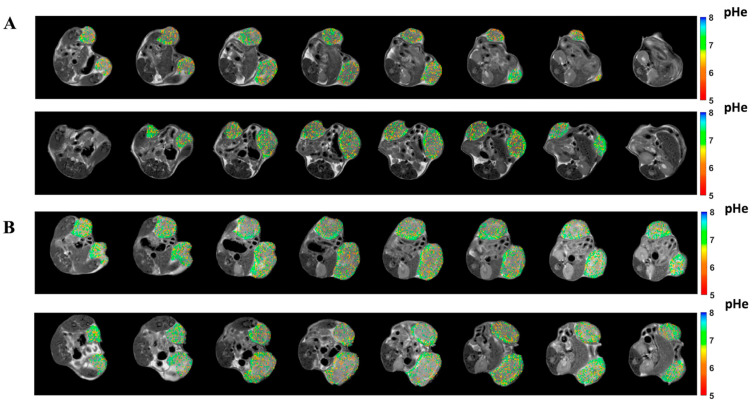
Representative pH images of control (**first row**) and Lansoprazole-treated (**second-row**) mice (from **top** to **bottom**) after 1 week (**A**) and after 2 weeks (**B**) post treatment, respectively.

A further small increase in the extracellular pH was observed after 2 weeks of treatment with Lansoprazole-treated mice in comparison to control ones (pHe = 6.89 ± 0.04 and 6.77 ± 0.04, *p* < 0.001, Figure 6A). Tumor pH images for representative mice show an increased number of pixels with less acidic pH values in Lansoprazole-treated tumor pH maps in comparison to control ones after 1 and 2 weeks of treatment (Figure 7A,B). 

To more precisely assess the spatial heterogeneity of extracellular pH inside the tumor region, the acidity score was calculated. A marked and statistically significant difference was observed in the acidity score between control and Lansoprazole-treated mice after 1 week of treatment (2.05 ± 0.05 and 1.94 ± 0.02, *p* < 0.001, Figure 6B) and after 2 weeks of treatment (2.04 ± 0.07 and 1.84 ± 0.07, *p* < 0.001, Figure 6B). Representative acidity score maps for control and Lansoprazole-treated mice after 1 and 2 weeks of treatment are shown in Supplementary Figure S4.

### 3.6. Amiloride Exhibits In Vivo Any Anti-Tumoral Effect and Is Ineffective in Altering Tumor pHe

A second cohort of mice was treated intra-peritoneally with Amiloride (5 mg/kg daily) when the tumor became palpable. No effect was observed on tumor growth either after 1 week or 2 weeks of treatment (Supplementary Figure S5). Amiloride treatment was ineffective in altering tumor acidosis; in fact, comparable extracellular tumor pH and acidity score values between control and treated mice at both time points were found (Supplementary Figure S6). Representative tumor pH images after 1 week and 2 weeks of treatment showed similar overall tumor size and tumor acidosis for the two groups (Supplementary Figure S7).

## 4. Discussion

In the present study, PPIs reduced cell viability in the two investigated aggressive breast cancer cell lines, with different potencies according to the drug and to the pH of the cell culture media. The obtained results indicated the promising therapeutic effect of Lansoprazole in inducing marked cytotoxicity and extracellular pH alteration in in vitro experiments, associated with a strong tumor growth inhibition in the case of the TNBC 4T1 murine model. Our study provided the first evidence that the in vivo anti-tumoral effect of Lansoprazole is associated with a significant increase in the tumor extracellular pH as measured through MRI-CEST pH imaging.

Tumor pH regulation is considered an important factor in promoting solid tumor growth and invasiveness through the involvement of several proton transporters. This results in conferring a selective advantage to cancer cells to survive in hypoxic and acidic microenvironments [14,71]. Several types of transmembrane transporters have been identified to have a role in determining the pH gradient between the intra- and the extra-cellular compartment, namely H^+^/K^+^-ATPase, Na^+^/H^+^ exchanger (NHE), vacuolar ATPase (V-ATPase), monocarboxylate transporters (MCTs), and carbonic anhydrase (CAs). These findings promoted the exploitation of several inhibitors as potential anticancer drugs [29,30]. On this basis, several studies investigated the efficacy of PPIs against breast cancers murine models. Lansoprazole has been studied in several murine and human breast cancer cell lines, showing marked cytosolic acidification and cytotoxicity, although most of the studies were limited to in vitro experiments or to the evaluation of the modulation of the activity when combined with doxorubicin or with cisplatin [38,72]. Our results are in agreement with these previous studies, showing that a marked cytotoxicity is achieved with concentrations above 100 µM. Of note, few studies investigated the anti-tumoral effect of Lansoprazole alone in breast cancer murine models, showing some efficacy in inhibiting tumor growth in the human breast cancer murine model [37]. However, none of them assessed in vivo the effect on the intra-/extra-cellular tumor pH. 

In our study, we found that only Lansoprazole was effective in delaying tumor growth and this efficacy was associated with a marked and prolonged reduction in the extracellular tumor acidification, demonstrating the in vivo inhibitory effectiveness of this drug. Recently, another V-ATPase inhibitor, Esomeprazole, demonstrated a remarkable reduced tumor growth and increased survival in the 4T1 TNBC murine model, although tumor pH was not evaluated [73].

Amiloride, an NHE1 inhibitor, has been shown to provide a moderate cell toxicity in human melanoma cancer cells, although it was ineffective in delaying melanoma tumor growth [74]. In breast cancer cells, only Amiloride derivatives have been investigated, with a modest cell toxicity in both murine and human cell lines, but a single treatment with this Amiloride derivative (EIPA) was less effective in reducing the tumor growth rate than the treatment with cisplatin alone [73,75]. Also in the present study, mice displayed no detectable reduction in tumor growth rate, although a moderate toxicity was observed in cellular experiments. 

Combined therapies where conventional cytotoxic agents are administered together or after a pretreatment with PPIs have been proposed as a strategy to overcome the chemo-resistance of several solid tumors caused by the acidic extracellular tumor microenvironment that reduces the intracellular accumulation of these drugs [76]. However, most of these therapies have demonstrated jeopardized treatment efficacy that appears dependent on the specific tumor type, dosage, and combination. One explanation for this reduced therapeutic potency arises from the inability to accurately measure the efficacy of these PPIs to modulate the acidic tumor microenvironment that, in turn, will affect the activity of the cytotoxic agents. We think that the in vivo imaging technique applied herein for precise measurements of the tumor extracellular pH is very useful to provide an accurate and reliable readout of the effect of PPIs. In fact, by providing precise measurements of the efficacy of PPIs to modulate tumor acidosis, one can provide a rationale whether such combo therapies can be successful (i.e., upon significant alteration of tumor pH) or ineffective (when the PPIs are not able to modify the acidity of the tumor microenvironment). Moreover, by accurately measuring tumor acidosis, the proposed method allows for seeking for the most efficient dose or treatment regimen as a function of the ability of the considered PPI to modify the extracellular pH for a given tumor type. The usefulness and the reliability of MRI-CEST tumor pH imaging has already been demonstrated in several tumor types upon treatment with a number of PPIs and alkalizer therapies [61,62,77].

## 5. Conclusions

The use of Proton Pump Inhibitors in breast cancer is an evolving area of research and holds promise for impacting the disease progression, especially in the context of metastasis, chemotherapy enhancement, and inflammation reduction [76,78,79]. However, significant research, including clinical trials, is required to determine the safety, efficacy, and optimal applications of PPIs in breast cancer therapy. Future perspectives on the use of PPIs in breast cancer remains promising but necessitate their precise role and optimal use in oncology [80,81,82]. MRI-CEST tumor pH imaging is an innovative imaging technique with the potential to provide valuable insights into the metabolic characteristics of breast cancer. Its non-invasive nature, ability to detect tumor acidosis, and application in early diagnosis and treatment monitoring make it a promising tool for improving breast cancer management in both preclinical research and clinical settings.

In conclusion, in vivo imaging of tumor acidosis can accurately provide an early readout of the therapeutic efficacy of PPIs in triple-negative breast cancers.

## Data Availability

All data generated or analyzed during this study are included in this published article. The raw data used and/or analyzed during the current study are available from the corresponding author on reasonable request.

## Supplementary Materials

The following supporting information can be downloaded at https://www.mdpi.com/article/10.3390/metabo13111161/s1, Figure S1: Cell viability results for TS/A breast cancer cells; Figure S2: Original WB for V-ATPase; Figure S3: Quantification of necrotic areas in 4T1 tumor sections; Figure S4: Acidity score maps of control and Lansoprazole-treated mice; Figure S5: Effect of Amiloride on tumor growth in vivo; Figure S6: Effect of Amiloride on extracellular tumor pH in vivo; Figure S7: Tumor pH maps for 4T1 mice treated with Amiloride.
