# Supplementary material for: Assessing the Therapeutic Efficacy of Proton Transport Inhibitors in a Triple-Negative Breast Cancer Murine Model with Magnetic Resonance Imaging—Chemical Exchange Saturation Transfer Tumor pH Imaging
Source: Metabolites. 2023 Nov 18;13(11):1161. doi: 10.3390/metabo13111161 (PMC10673543; doi:10.3390/metabo13111161)
Supplement: Supplementary file 1 [file metabolites-13-01161-s001.zip › metabolites-2681235-supplementary.pdf]

metabolites

SUPPLEMENTARY MATERIAL

Assessing the Therapeutic Efficacy of Proton Transport Inhibitors in a Triple-Negative Breast Cancer Murine Model with Magnetic Resonance Imaging–Chemical Exchange Saturation Transfer Tumor pH Imaging

Chetan Dhakan [1], Annasofia Anemone [2], Vittoria Ventura [2], Antonella Carella [1], Alessia Corrado [1], Elisa Pirotta [1], Daisy Villano [2], Feriel Romdhane [1], Francesco Gammaraccio [2], Silvio Aime [3] and Dario Livio Longo [1,*]

[1] Institute of Biostructures and Bioimaging (IBB), National Research Council of Italy (CNR), Via Nizza 52,
10126 Turin, Italy; chetandhakan09@gmail.com (C.D.); antonella.carella@unito.it (A.C.); alessia.corrado@unito.it (A.C.); elisa.pirotta@unito.it (E.P.); feriel.ramdhane@unito.it (F.R.)
[2] Department of Molecular Biotechnology and Health Sciences, University of Turin, Via Nizza 52, 10126 Turin, Italy;
annasofia.anemone@gmail.com (A.A.); vittoriaventura@hotmail.it (V.V.); villanodaisy@gmail.com (D.V.); francesco.gammaraccio@unito.it (F.G.)
[3] IRCCS SynLAB SDN , Via Gianturco 113, 80143 Naples, Italy; silvio.aime@unito.it
* Correspondence: dariolivio.longo@cnr.it

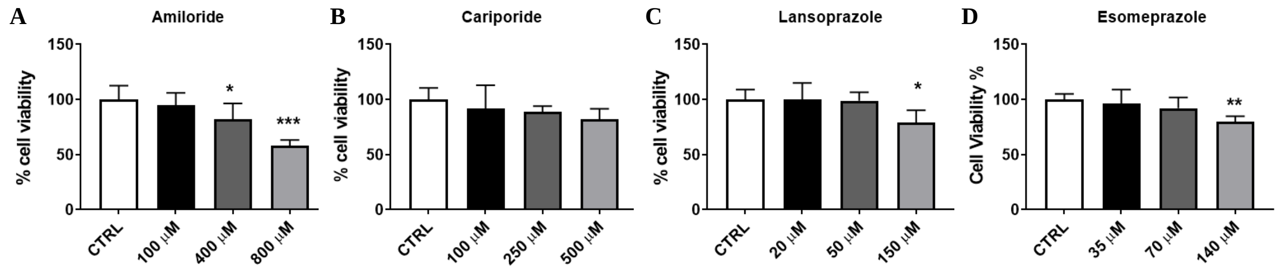

Figure S1. MTT results for TS/A murine breast cancer cell line in normoxia condition at 24 hours after treatment with Amiloride, Cariporide, Lansoprazole and Esomeprazole at several concentrations. All drug concentrations are expressed in µM. The statistic differences resulted from ANOVA analysis with Bonferroni post-hoc correction against the control group (* *p value* < 0.05; ** *p value* < 0.01; *** *p value* < 0.001).

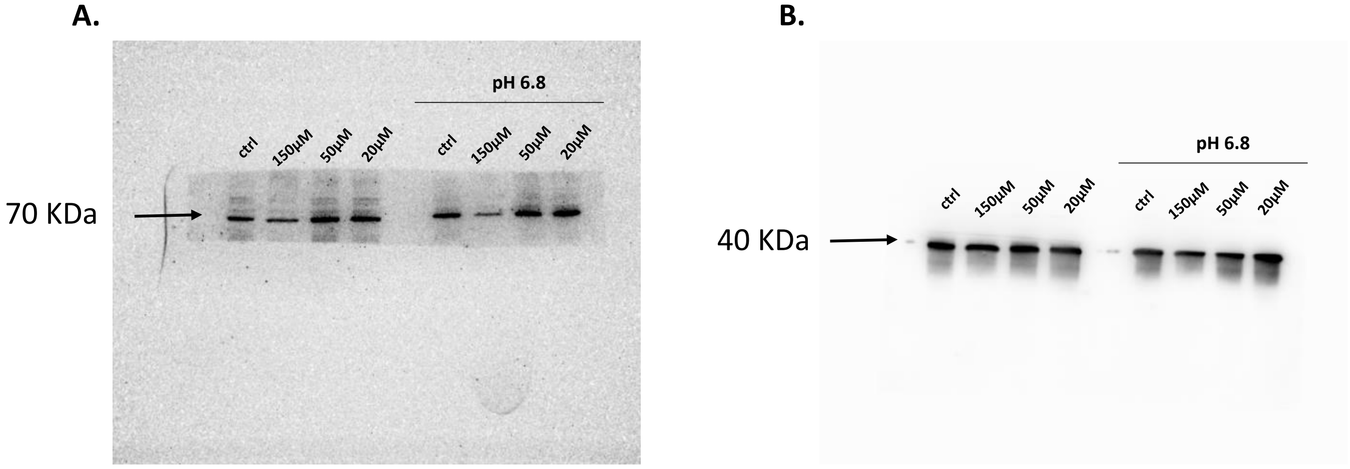

Figure S2. Original WB pictures for V-ATPase (**A**) and actin (**B**) proteins of 4T1 cells at pH = 7.4 and for acid-adapted 4T1 cells after 24h Lansoprazole treatment (150 µM-50 µM-20 µM).

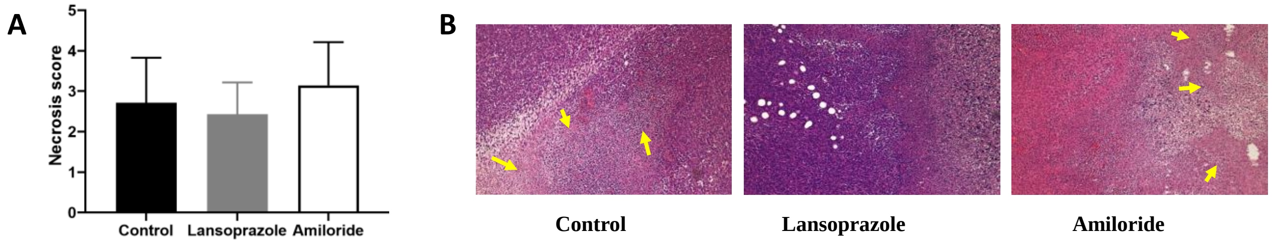

Figure S3. Quantification of necrotic areas in 4T1 tumor sections (n = 6) display a similar percentage of necrosis after treatment (**A**). Representative haematoxylin and eosin sections images of 4T1 tumor bearing mice for control, Lansoprazole and Amiloride treated groups immediately after last imaging session: yellow arrows indicating necrotic areas (**B**).

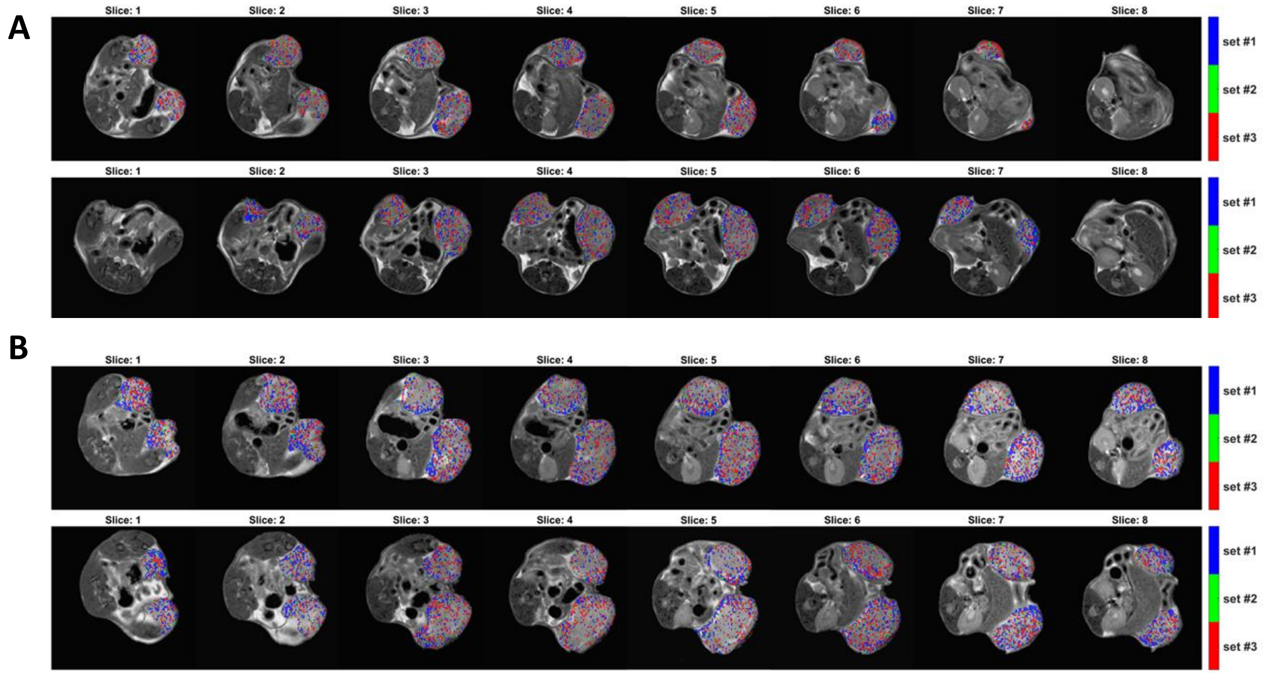

Figure S4. Representative acidity score maps of control (top) and Lansoprazole-treated mice (bottom) after 1 week (**A**) and after 2 weeks (**B**) post treatment, respectively.

metabolites

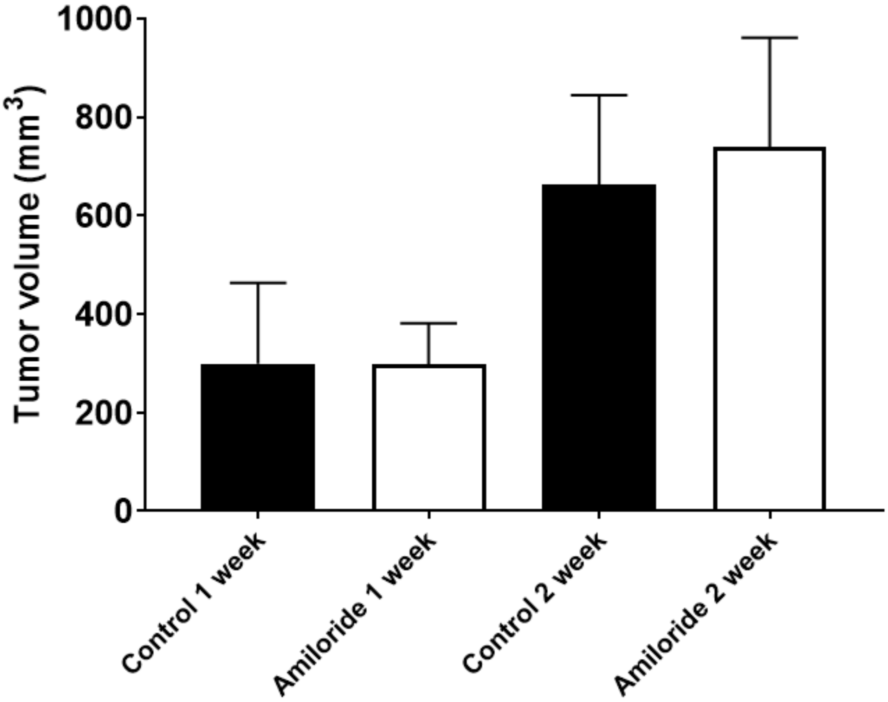

Figure S5. Effect of Amiloride (NHE1 inhibitor) on tumor growth in vivo. Measured tumor volumes for control and Amiloride-treated mice (5 mg/kg daily, i.p.) upon 1 week and 2 weeks of treatment.

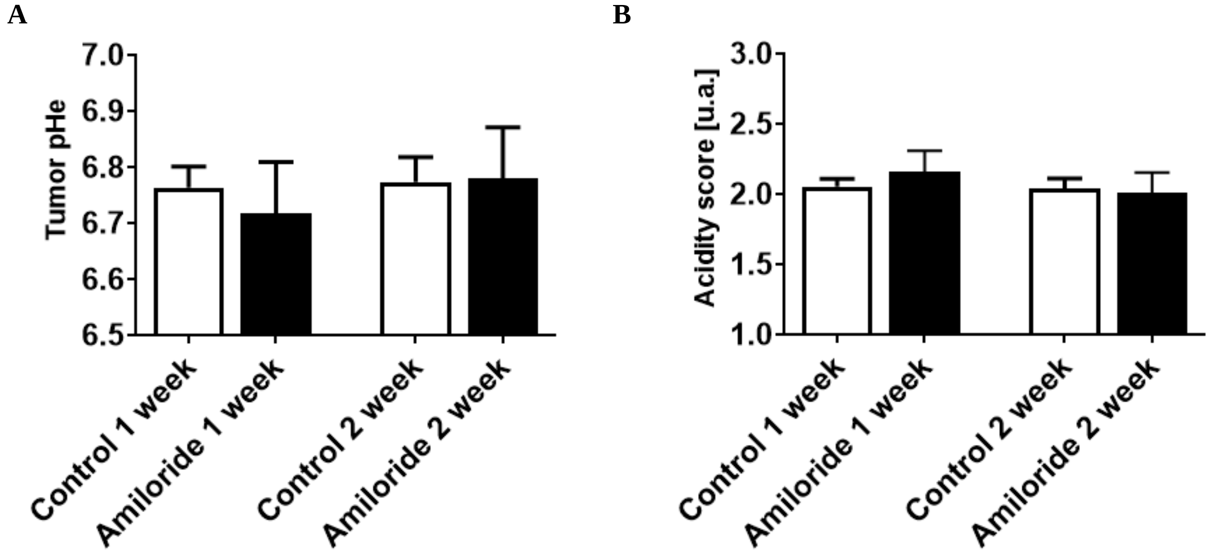

Figure 6: Effect of Amiloride (NHE1 inhibitor) on extracellular tumor pH (**A**) and on the acidity score values (**B**) upon 1 week and 2 weeks of treatment.

metabolites

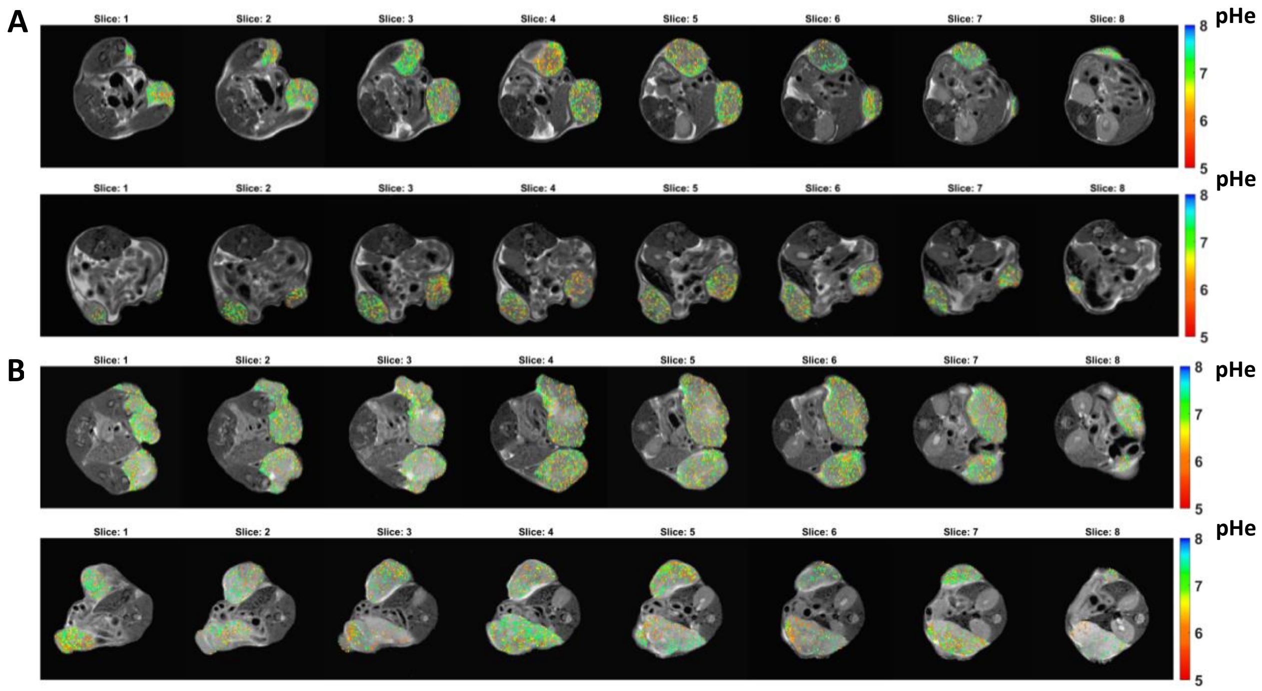

Figure S7. Representative tumor pHe maps overlaid to anatomical T2w images of control and Amiloride-treated mice (from top to bottom) after 1 week (**A**) and after 2 weeks (**B**) post treatment, respectively.
